# Genome-Wide DNA Methylation Analysis Reveals Epigenetic Signatures of Early Life Stress in Major Depressive Disorder Patients

**DOI:** 10.1192/j.eurpsy.2026.10609

**Published:** 2026-07-31

**Authors:** Y. Dwivedi, A. Francis, B. R. Roy, R. Shelton

**Affiliations:** Psychiatry, University of Alabama at Birmingham, Birmingham, United States

## Abstract

**Introduction:**

Major Depressive Disorder **(**MDD) is a pervasive condition, affecting over 20% of the global population. ELS, encompassing events like abuse and neglect, is a well-documented risk factor for the development of MDD in later life. A significant dimension of this relationship involves epigenetic modifications, which are increasingly recognized as key mechanisms through which environmental exposures, such as ELS, interact with an individual’s genetic predispositions to shape the depression phenotype.

**Objectives:**

The present study investigated genome-wide DNA methylation profiles in individuals with MDD, stratified by ELS exposure, and included trauma-matched control groups.

**Methods:**

The participants included 90 male and female adult patients aged 18-65 who met DSM-5 diagnostic criteria for MDD or volunteer controls as determined by the MINI International Neuropsychiatric Interview. Subjects comprised of MDD with ELS (n = 30), MDD without ELS (n = 30), and non-psychiatric controls with and without ELS (n = 30). The UAB IRB approved the protocol, and written informed consent was obtained from all participants. Early life trauma was assessed using the CTQ. Genome-wide methylation was done using reduced representation bisulfite sequencing. Differential methylation was evaluated by statistical tests conducted at each CpG location to determine significance, subsequently followed by the identification of differentially methylated regions (DMRs). The omics annotations derived from the ANNOVAR framework were integrated to determine biological implications and the CNS trait association of the DMR genes. To explore the functional relevance of genes associated with the DMRs, GENE2FUNC module in the Functional Mapping and Annotation (FUMA) platform was used.

**Results:**

We identified 128 DMRs in MDD *vs.* control, 29 in MDD+ELS *vs.* control+ELS, and 13 in MDD non-ELS *vs.* control non-ELS comparisons. Notable genes exhibiting DMRs in MDD+ELS included *LONRF1*, *MAPK8IP1*, *SELENOO*, *PDXP,* and *CNNM3,* showing strong enrichment in brain-related tissues and regulatory elements. Functional enrichment highlighted MAPK, synaptic vesicle, and JAK-STAT pathways. In contrast, MDD non-ELS DMRs included genes such as *BCL7B*, *BLCAP*, and *NNAT*, which exhibited elevated expression in multiple brain regions and were enriched in Hippo, Wnt, and Ras signaling pathways and chromatin remodeling.

**Conclusions:**

This study provides compelling evidence of distinct DNA methylation signatures associated with ELS-induced MDD and MDD without ELS exposure, highlighting the complex interplay between genetic and environmental factors in the etiology of this complex disorder. These results contribute to our understanding of the molecular mechanisms underlying ELS-induced MDD and may have implications for the development of novel diagnostic and therapeutic strategies.

**Disclosure of Interest:**

None Declared

